# SMOKING BEHAVIOUR: PATTERNS AND COSTS OF HEALTH SERVICE USAGE USING HAGIS AND LINKED ADMINISTRATIVE HEALTH DATA

**DOI:** 10.1093/geroni/igz038.3429

**Published:** 2019-11-08

**Authors:** Elaine Douglas, David Bell

**Affiliations:** 1 University of Stirling, Stirling, Scotland, United Kingdom; 2 University of Stirling, Stirling, United Kingdom

## Abstract

The associations between smoking and health are well documented. Using the Healthy Ageing In Scotland (HAGIS) survey linked to the administrative Scottish National Health Service (NHS) records this study analyses health service resource usage by older people according to self-reported smoking status. Individual level smoking status (current, ex-smoker, or never smoked), socio-demographic characteristics (age, gender, level of deprivation) and subjective health are sourced from people aged 50+ across Scotland using HAGIS. These responses are then linked to NHS Scottish Morbidity Records to analyse variation in health service usage as measured by the total number of days spent in hospital (daycases and inpatient stays), number of stays, and mean length of stay. Costs are then assigned by medical speciality. We use a two-part model to analyse the i) the probability of having been hospitalised at all, and ii) the quantum of resource usage and its associated cost for those who have been in hospital. Our study provides a conceptual and empirical framework for the associative relationship between smoking status and actual (rather than self-reported) health service usage and expenditure. This study demonstrates the insights to be gained from the linkage of individual survey responses to administrative health service data on resource usage and costs, and discusses the implications for health policy.

